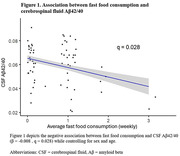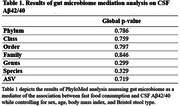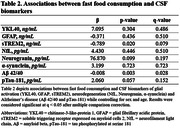# Investigating the role of gut microbiome in associations between fast food consumption and cerebrospinal fluid biomarkers of Alzheimer's disease in a cognitively unimpaired cohort

**DOI:** 10.1002/alz.086619

**Published:** 2025-01-09

**Authors:** Darby Peter, Margo B. Heston, Qilin Hong, Puja Agarwal, Tyler K. Ulland, Sterling C. Johnson, Sanjay Asthana, Rob Knight, Rima Kaddurah‐Daouk, Henrik Zetterberg, Kaj Blennow, Federico E. Rey, Barbara B. Bendlin

**Affiliations:** ^1^ Neuroscience Training Program, University of Wisconsin‐Madison, School of Medicine and Public Health, Madison, WI USA; ^2^ Wisconsin Alzheimer's Disease Research Center, University of Wisconsin School of Medicine and Public Health, Madison, WI USA; ^3^ Department of Medicine, University of Wisconsin‐Madison School of Medicine and Public Health, Madison, WI USA; ^4^ Department of Biostatistics and Medical Informatics, University of Wisconsin‐Madison, Madison, WI USA; ^5^ Rush Alzheimer's Disease Center, Rush University Medical Center, Chicago, IL USA; ^6^ University of Wisconsin School of Medicine and Public Health, Madison, WI USA; ^7^ University of Wisconsin‐Madison School of Medicine and Public Health, Madison, WI USA; ^8^ Center for Microbiome Innovation, University of California San Diego, La Jolla, CA USA; ^9^ Department of Medicine, Duke University, Durham, NC USA; ^10^ Institute of Neuroscience and Physiology, Department of Psychiatry and Neurochemistry, The Sahlgrenska Academy, University of Gothenburg, Mölndal, Gothenburg Sweden; ^11^ Institute of Neuroscience and Physiology, Department of Psychiatry and Neurochemistry, The Sahlgrenska Academy, University of Gothenburg, Mölndal Sweden; ^12^ Department of Bacteriology, University of Wisconsin‐Madison, Madison, WI USA; ^13^ Neuroscience Training Program, University of Wisconsin‐Madison, Madison, WI USA

## Abstract

**Background:**

Emerging evidence underscores the significant influence of diet on risk for Alzheimer’s disease and related dementias (ADRD). In particular, a Western dietary pattern associates with increased risk for ADRD, with proposed mediation via inflammatory mechanisms, among others. Although a Western dietary pattern associates with gut microbiome alterations, it remains unclear whether microbial alterations mediate Western diet‐associated inflammation and neurodegeneration. To begin to investigate these relationships, this study assessed whether the gut microbiome mediates associations between fast food consumption and cerebrospinal fluid (CSF) biomarkers of neurodegeneration, glial activation, and Alzheimer’s disease (AD).

**Method:**

Cognitively unimpaired adults (n=86, Wisconsin Registry for Alzheimer’s Prevention and Wisconsin Alzheimer’s Disease Research Center) underwent lumbar puncture, provided a stool sample, and reported average weekly fast food consumption over the past year via questionnaire. Participants were an average of 67 years of age and 62% of participants (n = 53) were female. Fecal microbiome composition was characterized using 16S rRNA sequencing. A QIIME2/Phyloseq pipeline was used to complete denoising, feature classification, filtration of rare taxa and agglomeration at each taxonomic rank. CSF biomarkers were quantified using the NeuroToolKit panel of robust prototype assays (Roche Diagnostics International Ltd, Rotkreuz, Switzerland). Multiple regression tested associations between fast food consumption and CSF biomarkers of neurodegeneration, glial activation, and AD. Phylogenetically‐informed mediation (PhyloMed; Hong et al, 2021) was performed at each taxonomic rank to identify bacterial clades mediating the relationship between fast food and the CSF Aβ42/40 amyloid pathology test.

**Result:**

A significant association between fast food and CSF Aβ42/40 was observed, such that more frequent fast food consumption associated with lower levels of CSF Aβ42/40 (Figure 1), but no significant mediating effects of gut microbiome were observed (Table 1). No significant associations between fast food consumption and other CSF biomarkers were observed (Table 2).

**Conclusion:**

While associations between fast food consumption and CSF biomarkers were expected to be partially mediated by gut microbial alterations, few individuals reported consuming fast food more than once each week. Further evidence is needed to characterize the influence of fast food and its nutritional components on neurodegeneration and development of AD pathology preclinically.